# Plasma Glycated CD59 and Gestational Diabetes Mellitus: A Systematic Review

**DOI:** 10.1002/edm2.70013

**Published:** 2024-11-15

**Authors:** Zahra Asadi, Roya Safari‐Faramani, Faranak Aghaz, Asad Vaisi‐Raygani, Saba Jalilian

**Affiliations:** ^1^ Students Research Committee Kermanshah University of Medical Sciences Kermanshah Iran; ^2^ Department of Clinical Biochemistry, Medical School Kermanshah University of Medical Sciences Kermanshah Iran; ^3^ Research Center for Environmental Determinants of Health, School of Public Health Kermanshah University of Medical Sciences Kermanshah Iran; ^4^ Nano Drug Delivery Research Center, Health Technology Institute Kermanshah University of Medical Sciences Kermanshah Iran; ^5^ Fertility and Infertility Research Center, Health Technology Institute Kermanshah University of Medical Sciences Kermanshah Iran

**Keywords:** adverse pregnancy outcomes, biomarker, gestational diabetes mellitus, large for gestational age, pGCD59, postpartum glucose intolerance

## Abstract

**Aims:**

Gestational diabetes mellitus (GDM) is a common complication of pregnancy worldwide. The standard method for screening GDM is the 75 g oral glucose tolerance test (OGTT). However, the OGTT is difficult, time‐consuming and requires fasting, making it an inconvenient test for GDM. Researchers have turned their attention to alternative biomarkers for GDM. This study aimed to systematically investigate the potential of plasma glycated CD59 (pGCD59) as a new biomarker for GDM and its associated adverse pregnancy outcomes.

**Methods:**

The systematic review was performed in the PubMed, ISI Web of Science, Scopus and Google Scholar databases from 1/1/2000 to 4/1/2024, and relevant studies were selected based on the inclusion and exclusion criteria. The quality of the studies was assessed using the Newcastle‐Ottawa scale.

**Results:**

The study revealed that pGCD59 levels before 20 weeks and during the second trimester of pregnancy have the potential to predict the results of the OGTT and also forecast adverse pregnancy outcomes, such as postpartum glucose intolerance (PP GI), neonatal hypoglycaemia (NH) and having large for gestational age (LGA) infants. The predictive ability of pGCD59 was found to be affected by the GDM status, especially body mass index (BMI).

**Conclusions:**

In conclusion, pGCD59 may be a promising indicator of glucose levels and could serve as a new biomarker for GDM. However, additional studies are needed to establish a reliable reference range and cut‐off value for pGCD59.

## Graphical abstract

The levels of pGCD59 have the potential to predict the results of OGTT. Certain adverse pregnancy outcomes can be anticipated using pGCD59. GDM status influences the predictive ability of pGCD59. The glucose level is better indicated by pGCD59 than by the glucose tolerance test.

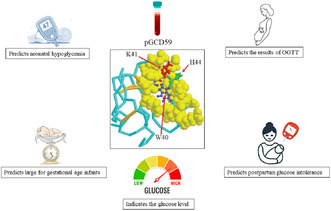

## 1 Introduction

Although most pregnancies proceed without any incident, complications can threaten the health of both the mother and foetus in some pregnancies [1]. Gestational diabetes mellitus (GDM) is a common metabolic disorder that affects 14% of pregnancies worldwide and is first diagnosed during pregnancy in women without a history of diabetes [2].

GDM can increase the risk of various adverse pregnancy outcomes such as caesarean section delivery, cardiovascular disease, metabolic syndrome, renal disease [3], polyhydramnios [4], hypertensive disorders of pregnancy (HDP), vascular ruptures and placental abruption [5]. In addition, women with a history of GDM are at a higher risk of developing glucose intolerance after childbirth. They have a tenfold higher chance of developing type 2 diabetes (T2DM) or prediabetes after delivery compared to women without a history of GDM [6]. Approximately 50% of women who experience persistent glucose intolerance in early postpartum (impaired fasting glucose and/or impaired glucose tolerance) develop T2DM within 5 years after giving birth [7].

Moreover, GDM can lead to various complications in foetuses and infants, including prematurity, future diabetes and obesity, large for gestational age (LGA) [8], shoulder dystocia, macrosomia [9], intrauterine growth restriction, impairment of nutrient circulation to the foetus [10] and neonatal intensive care (NICU) admissions [11]. Neonatal hypoglycaemia (NH) is a common complication in newborns of women with GDM, and it is characterised by low levels of glucose in the blood, below 2.6 mmol/L [12]. NH can result in brain damage, executive dysfunction and impairments in visual‐motor and neurodevelopmental processes [13].

Studies indicated that women who are diagnosed with GDM in early pregnancy, have a higher risk of developing adverse outcomes in the mother, foetus and offspring. It is hypothesised that early diagnosis of women at risk of or with GDM can reduce adverse pregnancy outcomes through applying risk‐modifying interventions, early treatment (metformin, insulin, etc.), regular follow‐up and monitoring, as well as lifestyle improvement through exercise and diet that contribute to a safe delivery [14, 15].

GDM is screened based on the World Health Organisation (WHO) criteria (2013) by the 75 g oral glucose tolerance test (OGTT) (fasting plasma glucose ≥ 5.1 mmol/L, and/or 1‐h plasma glucose ≥ 10.0 mmol/L, and/or 2‐h plasma glucose ≥ 8.6 mmol/L) at the end of the second trimester of pregnancy (24–28 weeks) [16]. A two‐step protocol of using a 1 h 50 g glucose challenge test (GCT) followed by a 3 h 100 g OGTT is conducted when the GCT fails [17]. OGTT has several limitations that restrict its use. It is not cost‐ or time‐effective and has poor reproducibility. The sample conditions can make an error in the glucose measurement, making it an unreliable test [18]. Additionally, OGTT requires fasting, and ingesting a glucose drink can cause vomiting or discomfort for some patients [19].

The reference range for glucose in GDM screening is unsuitable for early pregnancy since it cannot identify mothers at risk of developing GDM [20]. Additionally, postpartum (PP) glucose intolerance in GDM patients is determined by the OGTT test recommended by the American Diabetes Association (ADA) and performed 6–12 weeks after delivery [21]. However, some patients may decline to take this test due to physical restrictions or the unpleasant, difficult and time‐consuming nature of the OGTT [22]. Other tests, such as the measurement of glycated albumin/fructosamine or HbA1c, are not suitable for GDM screening due to their low specificity and sensitivity [23, 24]. So, there is a need for a sensitive, reproducible and easy‐to‐measure diagnostic biomarker that can be measured in a blood test without fasting. It must be able to identify women with GDM in early or late pregnancy and replace the PP glucose intolerance test.

CD59 is a glycosyl‐phosphatidylinositol‐anchored protein expressed on the membrane of mammalian cells. It regulates the complement system activation by preventing the formation of the membrane attack complex (MAC) on the self‐cells, inhibiting complement‐mediated cell lysis [25]. In diabetes, CD59 is inactivated by non‐enzymatic glycation on its glycation motif at amino acid residues K41‐H44, located in the core of the active site, around amino acid residue W40. This results in the formation of fructosyl‐lysine‐CD59 (glycated CD59 [GCD59]) [26]. This leads to the deposition of MAC on the cells and cell lysis. GCD59 can detach from the membrane, and its soluble form is found in urine and blood [27]. A highly sensitive and specific enzyme‐linked immunosorbent assay (ELISA) can measure the plasma‐glycated CD59 (pGCD59). The significantly higher levels of pGCD59 are found in individuals with type 2 diabetes mellitus (T2DM). pGCD59 is a new biomarker of hyperglycaemia and glucose intolerance (GI), predicting the response to OGTT. It can identify T2DM in a non‐fasting single blood test with high specificity and sensitivity [28, 29].

Thus, we undertook the present systematic literature review to establish whether pGCD59 is a reliable biomarker to predict GDM and its related adverse pregnancy outcomes.

## 2 Methods

This systematic review was conducted using the Preferred Reporting Items for Systematic Reviews and Meta‐Analyses (PRISMA) guidelines.

### 2.1 Search Strategy

The study aimed to find if pGCD59 is a good predictor for GMD and its related adverse pregnancy outcomes. A comprehensive search of PubMed, ISI Web of Science, Scopus and Google Scholar was conducted to achieve relevant studies from 1/1/2000 until 4/1/2024. The PICO formulation (P: patient or problems; I: intervention being considered; C: comparison intervention; O: outcome measurements) was applied. The keywords were used and selected from the Medical Subject Headings database. Table 1 describes the search terms used in this study. The reference lists were also manually checked to retrieve additional articles. The obtained studies were transferred to the EndNote (X9) reference manager.

**TABLE 1:** Search terms.

Databases: PubMed, ISI web of science, scopus and google scholar (until 4/1/2024)
#1 “CD59” OR “GCD59” OR “pGCD59” OR “Glycated CD59” #2 “Gestational diabetes mellitus” OR “GDM” OR “Pregnancy‐induced diabetes” OR “Pregnancy‐induced hyperglycemia” OR “Pregnancy‐induced glucose intolerance” #3 #1 AND #2

### 2.2 Study Selection and Eligibility Criteria

The search was conducted by two independent individuals (Zahra Asadi and Faranak Aghaz) and the contradictions created by a third person (Roya Safari‐Faramani) were investigated.

#### 2.2.1 Inclusion Criteria

The studies focused on patients with GDM rather than other types of diabetes, like pre‐pregnancy type 2 diabetes mellitus. GDM diagnosis in all included studies was based on a 2 h 75 g OGTT at 24–28 weeks according to the WHO 2013 criteria. The cohort and case‐control studies were included. The studies assessing the population who underwent the Vitamin D and Lifestyle Intervention (DALI) trial were considered too.

#### 2.2.2 Exclusion Criteria

This study excluded all non‐human, non‐English language studies, letters to editors, case reports, commentaries, conference abstracts, narrative reviews, editorials and duplicate publications. Protocols for prospective cohort studies were also excluded. Studies in which subjects had pre‐existing diabetes were not considered for inclusion. Moreover, studies that evaluated CD59 levels and those involving type 1 diabetes patients were not included in the systematic review.

### 2.3 Data Extraction and Synthesis

The title and abstracts of the extracted articles were screened by two authors (Z.A. and F.A.). The full texts were also assessed for the inclusion and exclusion criteria. The following data were manually extracted from each article: the author's name, the publication year, the type of study, participants, the sample size of case and control groups, body mass index (BMI), time of measurement, pGCD59 levels expressed as standard peptide units (SPU), the area under the curve (AUC) and the DALI trial.

### 2.4 Quality and Risk of Bias Assessment

The quality of each of the included articles (cohort and case‐control studies) was evaluated by the Newcastle‐Ottawa Scale (NOS), which assigns a maximum of nine stars from three aspects of selection (4 stars), study comparability (2 stars) and outcome measurement (3 stars) by two independent team members (Z.A. and R.S.‐F.). Contradictions were resolved by the third member (F.A.). Studies were categorised based on NOS scores: 0–3 as low quality, 4–6 as moderate quality and scores ≥ 7 as high quality [30]. The protocol of the systematic review was registered with the International Prospective Register of Systematic Reviews (PROSPERO) under registration number CRD42024552380.

## 3 Results

### 3.1 Search and Eligible Research Reports

**FIGURE 1:**
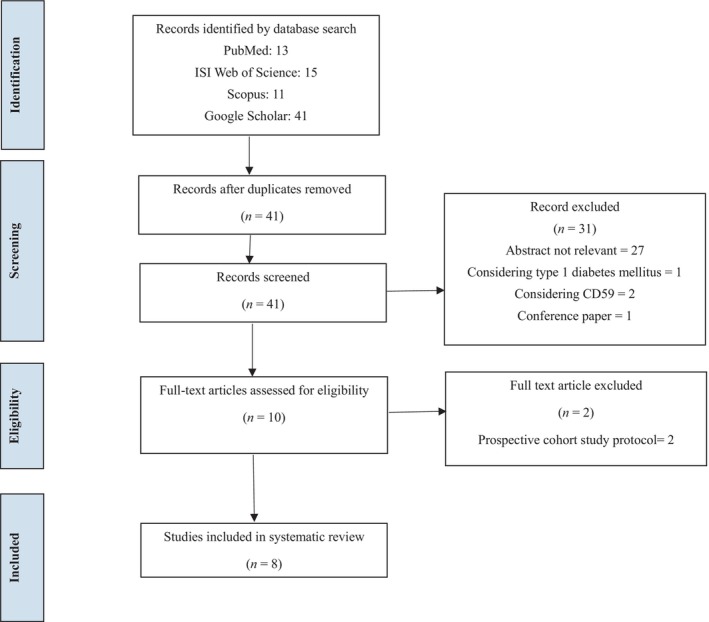
Flow chart of literature research.

A total of 80 studies were identified based on the inclusion criteria and were screened (Figure 1). After removing the duplicated studies, 41 papers were selected for a full‐text data review. Of these, 27 studies were considered irrelevant, one conference paper was excluded and three additional studies were eliminated because of consideration of type 1 diabetes (1) and CD59 (2). After thoroughly evaluating the full text of the 10 remaining articles, two studies provided a prospective cohort study protocol. Finally, eight studies were selected for full‐text data extraction.

The studies were published from July 2017 to November 2023. One was a case‐control study and seven were cohort studies. The levels of pGCD59 were measured by ELISA in all papers. Two studies investigated the pGCD59 as a biomarker for GDM [31, 32]. Other studies focused on the potential of pGCD59 in predicting GDM [33, 34], large for gestational age (LGA) newborns [34], adverse pregnancy outcomes [35], postpartum glucose intolerance [36, 37] and neonatal hypoglycaemia [38]. Table 2 represents the key characteristics of the studies included.

**TABLE 2:** Study characteristics.

Authors (year)	Type of Study	Participant		Sample size		BMI (kg/m^2^)	Time of measurement	pGCD59 of case (SPU)	pGCD59 of control (SPU)	AUC	DALI
		Case	Control	Case	Control						
Ma et al. (2020) [34]	Cohort	GDM	non‐GDM	207	486	≥ 29	< 20 weeks	3.9 ± 1.1	2.7 ± 0.7	0.86 (95% CI: 0.83–0.90)	Yes
Ghosh et al. (2017) [31]	Case–control	GDM	non‐GDM	127	500	Not reported	24–28 weeks	Median 10‐fold higher in GDM patients		0.92 (95% CI: 0.87, 0.96)	No
Bogdanet et al. (2023) [35]	Cohort	GDM	non‐GDM	103	275	28.7 (23.7–31.9)	24–28 weeks	2.6 (1.9–3.4)	2.39 (1.85–2.9)	0.81 (95% CI: 0.62–0.99	No
Bogdanet et al. (2022) [36]	Cohort	Women with GDM and PP GI	Women with GDM and normal PP GI	22	118	32.9 (27.8–35.8)	3 months PP	3.8 (2.8–4.2)	2.7 (2.2–3.7)	0.8 (95% CI: 0.70–0.91)	No
Bogdanet et al. (2022) [33]	Cohort	GDM	non‐GDM	103	275	28.7 (23.7–31.9)	< 14 weeks	3.7 (2.9–4.5)	3.6 (2.8–4.4)	0.63 (95% CI: 0.56–0.70)	No
Bogdanet et al. (2022) [38]	Cohort	Mothers of infants with NH	Mothers of infants without NH	30	369	≥ 29	< 20 weeks	3.2 ± 1.3	2.7 ± 0.8	0.70 (95% CI: 0.56–0.78)	Yes
Bogdanet et al. (2022) [32]	Cohort	GDM	non‐GDM	103	275	28.7 (23.7–31.9)	24–28 weeks	2.6 (1.9–3.4)	2.39 (1.85–2.9)	0.65 (95%CI: 0.58–0.71)	No
Benhalima et al. (2021) [37]	Cohort	Women with GDM and PP GI	Women with GDM and normal PP GI	35	70	27.0 ± 5.5	24–28 weeks	1.5 ± 0.6	1.0 ± 0.6	0.72 (95% CI 0.62–0.83)	No

Abbreviations: AUC, the area under the curve; BMI, body mass index; DALI, Vitamin D and Lifestyle Intervention; GDM, gestational diabetes mellitus; GI, glucose intolerance; NH, neonatal hypoglycaemia; PP, postpartum; SPU, standard peptide units.

### 3.2 Assessment of Risk of Bias

Table 3 displays the results of publication bias according to NOS. Three papers received 8 stars and 5 articles got 9 stars, indicating that these studies were high quality. Therefore, no study was excluded because of low‐quality scores.

**TABLE 3:** Details of the Newcastle‐Ottawa Scale score for included studies.

Study	Selection				Comparability	Outcomes			Total
	Representativeness of the exposed cohort	Selection of the non‐exposed cohort	Ascertainment of exposure	Demonstration that 0utcome of interest was not present at the start of study		Assessment of outcome	Was follow‐up long enough for outcomes to occur	Adequacy of follow‐up of cohorts	
34	*	*	*	*	*	*	*	*	8
31	*	*	*	*	**	*	*	*	9
35	*	*	*	*	**	*	*	*	9
36	*	*	*	*	**	*	*	*	9
33	*	*	*	*	**	*	*	*	9
38	*	*	*	*	*	*	*	*	8
32	*	*	*	*	**	*	*	*	9
37	*	*	*	*	*	*	*	*	8

## 4 Discussion

It has been indicated that in patients with T2DM, pGCD59 levels are higher than in healthy individuals, and it can predict the results of the OGTT [29]. The potential of pGCD59 as a biomarker for screening/diagnosing gestational diabetes was first proposed by Ghosh et al. in 2017. They found that in individuals with GDM based on the OGTT in the second trimester (T2), the levels of pGCD59 median were 10‐fold more than in non‐GDM women [31]. This difference was independent of covariates such as race, BMI, maternal and gestational age and multiplicity, with an adjusted AUC of 0.92. Women with pGCD59 values in the 6th decile or more had an eightfold higher likelihood of experiencing a failed GCT compared to those with values below the 6th decile [31]. Bogdanet et al. showed that the T2 pGCD59 was statistically higher in women with GDM with an AUC of 0.65 [32]. The Ghosh et al. study considers various study options, including screening tests with different diagnostic values, to identify GDM. The study also takes into account the existence of multiple pregnancies, which is associated with an increased risk of GDM [31, 39]. These factors help explain the different predictive abilities of pGCD59 observed in the studies.

T2 pGCD59 predicted different statuses of GDM based on fasting glucose, 1‐h glucose and 2‐h glucose. The difference in pGCD59 levels between the first and second trimesters (ΔpGCD59) was higher in the normal glucose tolerance group compared to the GDM group [32]. One explanation for this is that even in a normal pregnancy, there is a 16%–30% increase in blood glucose levels to supply the foetus with glucose, which is not considered GDM [40].

The GDM status of BMI subcategories also affected the predictive ability of T2 pGCD59, as the highest AUC values of 0.84 and 0.96 were observed at 35 kg/m^2^ ≤ BMI < 40 kg/m^2^ and BMI ≥ 40 kg/m^2^, respectively [32]. In a separate study, Bogdanet et al. discovered that a high BMI resulted in the highest AUC of 0.85 for BMI ≥ 35 kg/m^2^ and 0.88 for ≥ 40 kg/m^2^ for T1 pGCD59 [33]. Pre‐pregnancy BMI is associated with an increase in glucose intolerance, as a high BMI causes beta cell dysfunction and increases insulin resistance, leading to a higher risk of hyperglycaemia and pGCD59 formation [41].

Ma et al. conducted a study on the ability of pGCD59 to predict OGTT test results before 20 weeks of pregnancy in participants of the DALI study. The results showed that pGCD59 levels were higher in GDM patients than in non‐GDM cases in all maternal ages and ethnicities. pGCD59 accurately predicted the OGTT results at less than 20 weeks gestational age, with an AUC of 0.86. Moreover, < 20 pGCD59 predicted the results of patients with normal OGTT at less than 20 weeks who were later diagnosed with GDM in the second trimester [34] Generally, as a glycated protein, pGCD59 may reflect the glucose level and potentially be a biomarker.

It has been indicated that a history of GDM increases the risk of developing T2DM, especially within 5 years of PP. After being diagnosed with GDM, women are required to undergo the OGTT 6–12 weeks PP [42]. Bogdanet et al. and Benhalima et al. proposed the hypothesis that the PP pGCD59 levels can predict early PP GI in women with GDM based on the OGTT [36, 37]. Benhalima et al. found that early PP pGCD59 levels can predict GI test results with a cut‐off value of 0.9 SPU and moderate diagnostic accuracy [37]. In the Bogdanet et al. study, a cut‐off value of 1.9 SPU was obtained but was not significant across all categories of BMI, age and ethnicity. They recommended OGTT screening for women with pGCD59 > 1.9 SPU to detect PP GI [36]. An odd ratio (OR) of 3.3 for the development of GI was observed when PP pGCD59 increased by 0.5 units [37]. On the other hand, there was a correlation between a twofold increase in the incidence of PP GI and PP pGCD59 levels [36]. It seems that the performance of pGCD59 as a biomarker may vary depending on the degree of dysglycaemia, leading to different AUCs in various studies. The levels of pGCD59 and HbA1c at weeks 24–28 of pregnancy poorly predicted the development of early PP GI [37]. Therefore, pGCD59 in pregnancy cannot identify the women at risk of developing long‐term metabolic dysfunction. Compared with PP pGCD59, the accuracy of PP HbA1c in predicting the OGTT test was poor [37]. The increased erythropoietin production and decreased life span of red blood cells in pregnancy may make HbA1c an unsuitable marker for screening hyperglycaemia [43].

GDM is a risk factor for adverse pregnancy outcomes such as prematurely, large for gestational age (LGA), macrosomia, neonatal intensive care unit (NICU) admission, NH and metabolic disorders in newborns [44]. Besides, GDM causes adverse outcomes in mothers like both polyhydramnios and oligohydramnios [45]. Therefore, the potential ability of T1 and T2 pGCD59 to predict adverse pregnancy outcomes in women with GDM was assessed by Bogdanet et al. Women with polyhydramnios and oligohydramnios experienced higher and lower T2 pGCD59, respectively, with an excellent AUC (0.95) to predict the development of oligohydramnios [35]. The cause of polyhydramnios is not yet fully understood. However, foetal hyperglycaemia can lead to increased osmolarity and polyuria, which can cause polyhydramnios [46]. It is worth noting that the elevated T2 pGCD59 levels observed in women with polyhydramnios appear to be linked to the severity of hyperglycaemia, which is significant enough to cause this osmotic shift. Prematurely, admission to the NICU and PP jaundice were more common in infants born to mothers with GDM and higher ΔpGCD59 levels compared to mothers of healthy newborns [35].

Studies have shown a linear correlation between LGA prevalence and high levels of pGCD59. Women who delivered LGA infants had higher pGCD59 levels than women with non‐LGA infants even after considering maternal ethnicity, BMI and age [31, 34]. Besides, mothers of infants with macrosomia and LGA showed higher T1 pGCD59 levels compared with the mothers of healthy newborns with a better AUC than T2 pGCD59 [35]. In addition, 78% of LGA infants had mothers who were not diagnosed with GDM based on the OGTT but with a failed GCT. However, they had a 7‐fold higher T2 pGCD59 than the control group [31]. This is consistent with the previous studies, which showed that women with intermediate glucose tolerance are at a higher risk of abnormal pregnancy outcomes even with normal OGTT in the future [47].

NH as an adverse pregnancy outcome can be observed among newborns of women with GDM. The glucose transplacental transfer induces insulin secretion by the foetal pancreatic β‐cells, causing hyperinsulinaemia that begins from the first trimester of pregnancy. However, after delivery, when the glucose supply from the mother stops, the high insulin level can lead to neonatal hypoglycaemia (NH) [48]. The association between NH and levels of pGCD59 at < 20 weeks was assessed by Bogdanet et al. [38] Mothers of newborns with NH experienced significantly higher amounts of pGCD59. The prevalence of NH was higher among the infants of women with a BMI of 35 or more. For each 5‐unit increase in BMI, there was 60% higher odds of NH. The risk of developing NH was twice as high in infants born to mothers with pGCD59 values in tertile 3 (> 3.2) compared to the first tertile (≤ 2.5). As a result, maternal hyperglycaemia was correlated with NH and pGCD59, and the GDM status was a good predictor for NH [38].

## 5 Conclusion

This systematic review assessed the current evidence on GDM and pGCD59. The study found that pGCD59 levels were higher before week 20 and at T2 in GDM patients compared to women without GDM. The predictive ability of T1 and T2 pGCD59 was influenced by GDM status, with a high AUC in women with the highest BMI. It was also found that pGCD59 at T1 and T2 can predict adverse pregnancy outcomes in women with GDM. In addition, PP pGCD59 levels can predict early postpartum GI in women with GDM based on the OGTT. As a glycated protein, pGCD59 could reflect glucose levels, making it a potential biomarker for screening and diagnosis of gestational diabetes. In contrast to the time‐ and energy‐consuming OGTT, the pGCD59 test does not require fasting and can be measured via a simple blood sample with ELISA, ensuring optimal precision, reproducibility and clinical sensitivity. Although the test has not yet been commercially produced, ELISA procedures are standard in laboratories and do not require extensive training for qualified staff. However, the cut‐off values and reference intervals for pGCD59 have not yet been established and need further investigation. Additional research is also required to validate and standardise pGCD59 measurement before it can be recommended as a replacement for OGTT. Most studies have focused on the white population, highlighting the need for research involving a larger and more ethnically diverse cohort to evaluate the reliability of pGCD59 as a biomarker.

## Author Contributions

**Zahra Asadi:** study selection, data extraction and synthesis, quality and risk of bias assessment, writing – original draft preparation. **Roya Safari‐Faramani:** quality and risk of bias assessment, study selection, funding acquisition. **Faranak Aghaz:** database search, data extraction and synthesis, risk of bias assessment, writing – review and editing. **Asad Vaisi‐Raygani:** writing – review and revision. **Saba Jalilian:** database search, data extraction and synthesis.

## Conflicts of Interest

The authors declare no conflicts of interest.

## Data Availability

Data sharing is not applicable to this article as no new data were created or analyzed in this study.
